# Beyond Positive Response Rates: Capturing Information Richness in Workplace AI Acceptance Using Belief Structure TOPSIS

**DOI:** 10.3390/e28070759

**Published:** 2026-07-02

**Authors:** Ewa Roszkowska, Tomasz Wachowicz

**Affiliations:** 1 Faculty of Computer Science, Bialystok University of Technology, Wiejska 45A, 15-351 Bialystok, Poland; 2 Department of Operations Research, University of Economics in Katowice, 1 Maja 50, 40-287 Katowice, Poland; tomasz.wachowicz@uekat.pl

**Keywords:** multi-criteria analysis, survey data, information, belief structure, B-TOPSIS, uncertainty, workplace algorithms, artificial intelligence

## Abstract

This study applies the Belief Structure TOPSIS (B-TOPSIS) method to analyse cross-country attitudes toward AI-driven workplace practices across the EU27. The proposed approach preserves the full distribution of survey responses, explicitly incorporates uncertainty, and evaluates alternatives based on their distance from ideal and anti-ideal belief structures. Using data from Special Eurobarometer 554, we construct individual B-TOPSIS indexes for eight AI-related workplace applications and an aggregated B-TOPSIS index capturing overall acceptance. The results reveal systematic cross-country differentiation. Activities such as gathering applicant information, allocating work, and processing employee data generally receive moderate acceptance. Safety-focused applications are widely supported, whereas ethically sensitive practices, such as employee monitoring and automated dismissal, face low acceptance. Additionally, sensitivity analysis based on Monte Carlo simulation and stochastic dominance demonstrates that the obtained rankings remain highly stable under alternative assumptions regarding utility functions, confirming the robustness of the proposed framework. A comparison with rankings derived from total positive responses, commonly used in EU reports, shows that although the two approaches are strongly correlated, they are not interchangeable. By retaining the complete response structure, the proposed method captures differences in response intensity that are obscured by conventional summary measures. The findings highlight the multidimensional and conditional nature of workplace AI acceptance in the EU and demonstrate the value of belief-structure-based approach for analysing survey data.

## 1. Introduction

Digitalisation, artificial intelligence (AI), and algorithmic management technologies are becoming increasingly embedded in labour markets and organisational practices across the European Union (EU) [1,2]. Advances in data analytics, decision automation, and monitoring systems are reshaping recruitment, task allocation, supervision, and performance appraisal processes [3]. Recent research shows that AI-driven talent management platforms treat employees as data profiles, highlighting the broader socio-technical implications of AI adoption in organisations [4]. At the same time, these developments have intensified debates on job displacement, privacy, worker autonomy, fairness, and institutional accountability [5,6,7]. The European context is particularly relevant due to the EU’s regulatory ambitions, including the proposed AI Act [8], as well as its long-standing commitment to worker rights, procedural fairness, and data protection [9].

Recent survey initiatives, including OECD (2024) [10], ESENER (2024) [11], Eurobarometer 554 (2024) [12], EWCS (2024) [13], and AIM-WORK (2024–2025) [14], have begun to systematically document the adoption of AI systems and public attitudes toward their use in the workplace. However, comparative analyses often rely on descriptive indicators, such as the share of positive responses, which simplify ordinal response scales, collapse uncertainty (e.g., “do not know”), and flatten multidimensional response distributions. This reduction limits the analytical value of cross-country comparisons and may obscure meaningful differences in public attitudes toward workplace AI.

To address this limitation, the present study draws on individual-level microdata from Special Eurobarometer 554 [12] and applies the Belief Structure TOPSIS (B-TOPSIS) framework to analyse public attitudes toward AI-enabled workplace practices. The analysis covers key workplace activities in which AI is applied, including recruitment support, task allocation, data management, monitoring, performance evaluation, and managerial decision-making. Each activity is assessed using a four-point linguistic scale (very positive, somewhat positive, somewhat negative, very negative), complemented by a “do not know” category. Existing cross-country analyses based on survey data frequently employ aggregated indicators, such as total positive response rates, which are widely used in EU policy reports. While suitable for descriptive comparisons, these indicators compress the information contained in ordinal response distributions and may therefore overlook important differences between countries. To provide a benchmark against established practice, the present study reports results based on total positive response rates alongside those obtained from the B-TOPSIS framework.

The primary objective of this study is to develop a belief-structure-based framework for analysing cross-country differences among EU Member States. By evaluating alternatives in relation to positive and negative ideal belief solutions, B-TOPSIS [15,16,17] use the full response distribution and avoid the information reduction associated with aggregated response measures. This aligns with information-based approaches to decision optimisation under uncertainty in complex socio-technical systems.

In this study, the B-TOPSIS method is applied in two stages: first, to construct separate indexes for individual workplace AI activities, and then to build an aggregated multidimensional index of overall workplace AI acceptance across EU Member States.

Against this background, the study addresses three research questions:

- RQ1. How do citizens across EU Member States differ in their attitudes toward workplace AI applications?
- RQ2. How do EU Member States rank with respect to individual AI-related workplace activities when evaluated using the B-TOPSIS method?
- RQ3. How do EU Member States rank on an aggregated B-TOPSIS index capturing multidimensional attitudes toward workplace AI?

The answers for the aforementioned questions are particularly relevant for EU policy design, including the implementation of the AI Act [8], where workplace AI systems are classified as high-risk applications requiring enhanced governance and oversight. The findings may therefore inform debates on acceptable levels of automation in employment-related decision-making.

The contribution of this paper is threefold. Methodologically, it extends the application of B-TOPSIS to the analysis of AI-related attitudes, demonstrating the usefulness of belief-based approaches for analysing ordinal survey responses under uncertainty. The study also integrates Monte Carlo-based sensitivity analysis and stochastic dominance testing to evaluate the robustness of rankings under alternative utility assumptions. Empirically, it provides new comparative evidence on public acceptance of workplace AI across the EU-27 using ordinal survey microdata. Substantively, it offers both detailed and aggregated rankings that may support labour market policy, regulatory debates, and future assessments of AI-driven workplace transformation.

The remainder of the paper is structured as follows. Section 2 reviews the literature on AI and digital technologies in the workplace. Section 3 describes the data and the B-TOPSIS methodology. Section 4 presents the results of the individual and aggregated analyses. Section 5 discusses the implications of the findings, and Section 6 concludes by summarizing the main insights and suggesting directions for future research.

## 2. Literature Review—AI and Digital Technologies in the Workplace

### 2.1. Introduction

Artificial intelligence and digital technologies are increasingly transforming workplace practices, affecting recruitment, task allocation, monitoring, performance evaluation, and other HR processes. These developments are widely discussed not only in terms of efficiency gains but also in relation to fairness, transparency, autonomy, and employee well-being. The literature increasingly treats AI as a socio-technical phenomenon that reshapes workflows, governance structures, and workplace relations rather than as a purely technical tool [18,19,20].

In this study, the following literature review is not intended as an exhaustive or fully critical synthesis of empirical findings. Instead, it provides the conceptual and empirical background necessary to interpret and structure the analysis of Eurobarometer 554 data. For this reason, the reviewed studies are organised according to key workplace AI functions that directly correspond to the survey design and its response categories. The review focuses on the main domains of AI application in the workplace—namely, recruitment, task allocation, data management, employee monitoring, performance assessment, and managerial decision-making—to establish a consistent conceptual mapping between the literature and the empirical indicators used in the subsequent analysis of Eurobarometer 554 (2024) [12].

### 2.2. AI in Recruitment and Selection

AI tools such as chatbots, screening algorithms, and predictive analytics are increasingly used to improve recruitment efficiency and support candidate selection. Research suggests that these systems are most effective when they complement human decision-making by processing large volumes of applicant data and allowing HR professionals to focus on strategic and contextual judgments [18,19]. This reflects the concept of intelligence augmentation, where AI extends human cognitive capacity while ethical reasoning and holistic evaluation remain under human control, particularly in uncertain situations [18].

Empirical evidence supports these conclusions. AI-supported chatbots can improve interview efficiency and candidate comfort when they are well aligned with recruitment tasks [21]. Similar findings show that AI improves efficiency across the recruitment process, although concerns remain regarding implementation costs, privacy, and recruiter displacement [22]. Applications such as automated résumé screening, predictive analytics, and personalized communication also contribute to efficiency and cost reduction [23]. At the same time, AI may reduce bias in selection procedures, provided that systems are transparent and perceived as fair [24].

Overall, acceptance of AI in recruitment depends not only on efficiency gains but also on perceptions of fairness, transparency, and human oversight.

### 2.3. Task Allocation and Employee Management

AI systems are increasingly used in task allocation, scheduling, and workforce management. These technologies can improve efficiency by optimizing workload distribution and project assignments [18,19]. At the same time, their effects on employees depend heavily on how they are implemented and governed.

Research shows that AI may either enhance or constrain employee autonomy depending on whether it complements existing work, replaces tasks, or creates new functions [20]. Similar concerns emerge when AI capabilities do not align with worker expectations, increasing uncertainty and perceptions of job insecurity [25]. AI applications are also expanding across the employee lifecycle, influencing autonomy, organizational fairness, and internal coordination [26]. These changes are particularly visible in knowledge-intensive sectors, where digital systems increasingly shape the allocation of responsibilities and resources [27].

Empirical studies indicate that AI can improve engagement and efficiency, but extensive task substitution may also increase alienation and reduce employees’ sense of agency [28]. This has strengthened calls for participatory and ethically grounded AI governance frameworks [29].

AI-driven task allocation, therefore, affects not only efficiency but also the distribution of authority and control within organizations.

### 2.4. Data Management and Monitoring

AI-based systems enable organizations to collect, process, and store large amounts of employee data, improving monitoring, compliance, and workplace safety. However, these capabilities also raise concerns about privacy, surveillance, and the unequal distribution of informational power between employers and employees.

The literature identifies several ethical risks associated with AI monitoring, including reduced autonomy, weakened moral agency, and the normalization of surveillance practices [30]. Similar concerns are reported in AI-driven knowledge management systems, where privacy risks, bias, and weak mitigation strategies remain significant challenges [7]. Research on employee well-being management also shows that AI tools used to detect psychosocial risks may offer preventive benefits while intensifying ethical concerns related to surveillance [31].

To address the challenges associated with AI-based monitoring systems, the importance of transparency, accountability, participatory design, and data literacy is emphasized [6,32]. Even where AI systems improve personalization and analytical precision, unresolved concerns about privacy and ethics may undermine trust and acceptance [5].

These findings suggest that employee acceptance of AI-based monitoring depends less on technical performance than on governance mechanisms that ensure legitimacy, accountability, and trust.

### 2.5. Performance Assessment and Automated Managerial Decisions

AI technologies can improve the speed and consistency of performance assessment, but their use in evaluation and dismissal processes raises important ethical and regulatory concerns. While automation may increase productivity and efficiency, it may also intensify inequality and labour market disruption [33].

Research indicates that worker responses to AI-based managerial systems are mixed. Even in technologically advanced environments, concerns remain regarding fairness, leadership quality, and governance [34]. AI may either reinforce or reduce workplace inequalities depending on the institutional context in which it is implemented [35].

Trust in AI-based managerial decisions depends strongly on transparency and the presence of human oversight. This is particularly important in high-stakes decisions such as performance evaluation or dismissal, where procedural fairness and accountability are critical [24,25].

Acceptance of AI in this domain is therefore especially sensitive to the transparency, accountability, and reversibility of algorithmic decisions.

### 2.6. Empirical Surveys on AI in European Workplaces

Large-scale surveys provide empirical evidence that complements the theoretical literature. ESENER (2024) documents the adoption of AI-related technologies in workplace risk management but does not directly measure employee attitudes [11]. EWCS (2024) examines workers’ experiences with digital tools, monitoring, and autonomy, but does not systematically distinguish between traditional and AI-enabled systems [13]. Eurobarometer 554 (2024) directly measures perceptions of AI across workplace activities using a common ordinal scale [12], while AIM-Work (2024–2025) focuses on algorithmic management, fairness, and autonomy, although with more limited country coverage [14].

Findings across these surveys show that workers generally recognize the efficiency benefits of AI while expressing concerns regarding privacy, surveillance, fairness, and automated dismissals. Applications associated with efficiency and workplace safety tend to be viewed more positively, whereas those linked to monitoring or punitive decisions receive less support. Among these datasets, Eurobarometer 554 (2024) is particularly useful because it measures perceptions across multiple workplace functions using a common scale, providing a consistent basis for cross-country comparisons [12].

### 2.7. Overall Summary

Table 1 summarizes the main workplace functions identified in the literature and the key opportunities and concerns associated with AI adoption. The selected workplace AI applications reflect the structure of Eurobarometer 554 and capture key domains of algorithmic decision-making in employment contexts, including recruitment, task allocation, monitoring, and managerial decisions. These domains represent areas where AI adoption raises both efficiency opportunities and concerns related to privacy, autonomy, and fairness, making them suitable for measuring public attitudes.

This synthesis provides the conceptual basis for the empirical analysis that follows [12]. It also shows that worker attitudes toward AI are often ambivalent, reflecting trade-offs between expected efficiency gains and concerns about autonomy, fairness, and managerial control. While the substantive literature extensively discusses the social and organisational implications of AI in the workplace, a separate methodological gap remains in how survey-based evidence is aggregated and compared across countries. In particular, many policy reports rely on simple percentage-based indicators of positive responses, which do not fully capture the ordinal nature of survey data or the uncertainty embedded in neutral and “do not know” responses. This study addresses this gap by applying a belief-structure-based multi-criteria framework that preserves the full response distribution.

## 3. Materials and Methods

### 3.1. Data Source and Variables

The empirical analysis is based on microdata from Special Eurobarometer 554: Artificial Intelligence and the Future of Work, conducted between 25 April and 22 May 2024 in the 27 EU Member States [12,37]. The survey was commissioned by the European Commission’s Directorate-General for Employment, Social Affairs and Inclusion and carried out by the Verian Group. The final sample included 26,415 respondents, selected according to the standard Eurobarometer sampling design to ensure representativeness of the adult population in each Member State. Interviews were conducted face-to-face or via CAPI in respondents’ native languages.

The survey covers four thematic areas: general perceptions of digital technologies, digital skills, awareness of AI at work, and attitudes toward AI and algorithmic systems in the workplace. This study focuses on eight items (QB8.1–QB8.8) measuring attitudes toward AI applications in workplace management [12,37]:

Q1—Gathering additional information on applicants for a job (QB8.1)
Q2—Selecting applicants for a job (QB8.2)
Q3—Allocating tasks to workers or managing their working schedules and shifts (QB8.3)
Q4—Collecting, processing, and storing workers’ personal data (QB8.4)
Q5—Improving workers’ safety and security (QB8.5)
Q6—Monitoring workers (QB8.6)
Q7—Assessing workers’ performance (QB8.7)
Q8—Automatically firing workers (QB8.8)

Each item uses a five-category response scale: “very positive”, “somewhat positive”, “somewhat negative”, “very negative”, and “don’t know”. Together, these items capture attitudes toward workplace AI applications ranging from supportive functions to managerial control functions and provide the basis for the comparative analysis.

Descriptive distributions of responses by country and socio-demographic characteristics are reported in Appendix A (Table A1, Table A2, Table A3, Table A4, Table A5, Table A6, Table A7 and Table A8) and Appendix B (Table A9).

### 3.2. Methods

The Belief Structure (BS) model, developed in [38,39,40], underlies belief structure-based TOPSIS (B-TOPSIS), an extension of classical TOPSIS designed to address uncertainty in group decision-making [41]. By incorporating Fuzzy Belief Structures, the method also captures imprecise and ambiguous evaluations [42,43].

B-TOPSIS has been applied in areas such as risk analysis [43], transport planning [44], Sociological Game Theory [45], and ordinal survey evaluation [15,16,17,46], demonstrating its usefulness in complex multi-criteria decision-making problems. In this study, the method is used to analyse individual survey items and construct an aggregated ranking of workplace AI attitudes across EU Member States.

The procedure consists of the following steps.

Step 1. Defining the Problem and Constructing the Belief Decision Matrix

For the problem, we consider a set of objects $O=\{O_{1},O_{2},\ldots ,O_{m}\}$, which are evaluated by respondents using different criteria $C=\left\{C_{1},C_{2},\ldots ,C_{n}\right\}$.

We assume an ordinal scale with N categories $H_{1},H_{2},\ldots ,H_{N}$, where each category $H_{k}$ is preferred over $H_{k+1}$. We also include an additional option: “Don’t know/No answer/Refuses.” Survey responses for each alternative $O_{i}\;$ and criterion $C_{j}$ are represented as Belief Structure (BS) model:

(1) $$BS\left(S_{ij}\right)=\left\{\left(H_{k};\;\beta_{ijk}\right);\;k=1,\ldots ,N\right\}$$

which can be simplified to a vector,

(2) $$S_{ij}=\left[\beta_{ij1},\beta_{ij2},\ldots ,\beta_{ijN}\right].$$

Here, $\beta_{ijk}=\frac{n_{ijk}}{n_{ij}}$ represents the proportion of respondents selecting grade $H_{k}$. The total belief degrees satisfy $\sum_{k=1}^{N}\beta_{ijk}\leq 1$ with the remaining portion $\beta_{ijH}=1-\sum_{k=1}^{N}\beta_{ijk}$ representing the degree of ignorance, i.e., the proportion of respondents who chose “Don’t know,” “No Answer,” or “Refused.”

This yields the Belief Decision Matrix (BM):

(3) $$BM=[S_{ij}],$$

where each entry $S_{ij}$ represents the belief structure for criterion $C_{j}$ and object $O_{i}\;(i=1,2,\ldots m;\;j=1,2,\ldots ,n).$

This step converts ordinal survey data into a form suitable for MCDM.

Step 2. Normalizing the Belief Decision Matrix

If the BS model contains uncertain responses, the incomplete belief structures are normalized using the Proportion of Potential (POP) approach and its reduced form:

(4) $${\bar{S}}_{ij}=\left[\;\frac{\beta_{ij1}}{\sum_{k=1}^{N}\beta_{ijk}},\;\frac{\beta_{ij2}}{\sum_{k=1}^{N}\beta_{ijk}},\ldots ,\frac{\beta_{ijN}}{\sum_{k=1}^{N}\beta_{ijk}}\right]$$

The resulting vectors form the Normalized Belief Decision Matrix (NBM):

(5) $$NBM=[{\bar{S}}_{ij}].$$

This procedure redistributes uncertainty proportionally to the observed distribution, preserving the structure of the responses and appropriately scaling belief levels.

Step 3. Specifying the Utility Function and Similarity Structure

Each linguistic grade $H_{k}\;$ is mapped to a utility $U\left(H_{k}\right)\in [0,1]$, satisfying $0\leq U\left(H_{k}\right)\leq 1$. The similarity between grades is defined as:

(6) $${\tilde{s}}_{ij}\left(H_{i},H_{j}\right)=1-\left|U\left(H_{i}\right)-U\left(H_{j}\right)\right|$$

yielding the similarity matrix

(7) $$\tilde{S}=[{\tilde{s}}_{ij}],$$

where ${\tilde{s}}_{ij}$ denotes the similarity between grades $H_{i}$ and $H_{j}$.

This step embeds the ordinal scale into a continuous preference space.

Step 4. Weighting the Criteria

Weights $w_{j}$ are assigned to criteria, where $\sum_{j=1}^{n}w_{j}$.

Assigning weights in socio-economic multi-criteria analyses is challenging, as no universally accepted method exists. Common approaches include objective, subjective, and hybrid weighting schemes, and in the absence of empirical prioritisation, equal weights are typically used [47,48,49].

Step 5. Determining the Ideal and Anti-Ideal Belief Solutions

For each criterion, ideal and anti-ideal reference structures are defined as:

(8) $$S_{j}^{+}=\left[1,\ldots ,\;0\right],{\;S}_{j}^{-}=\left[0,\ldots ,\;1\right],\;\;\;j\;=\;1,\;2,\;\ldots ,\;n.$$

The Positive Ideal Belief Solution (PIBS) is:

(9) $$A^{+}=\{S_{1}^{+},\ldots ,S_{n}^{+}\}$$

where $S_{j}^{+}$ is the best belief structure model for the *j*-th criterion (j = 1, 2, ..., n).

The Negative Ideal Belief Solution (NIBS) $A^{-}$ is:

(10) $$A^{-}=\{S_{1}^{-},\ldots ,S_{n}^{-}\}$$

where $S_{j}^{-}$ is the worst belief structure model for the *j*-th criterion (j = 1, 2, ..., n).

Step 6. Computing the Separation Measures

The separation measure from BIBS, $D_{i}^{+}$ is given by:

(11) $${D_{i}^{+}=D}_{i}^{+}\left(O_{i},A^{+}\right)=\sqrt{\sum_{j=1}^{n}{w_{j}d_{BS}\left(\;\;{\bar{S}}_{ij},S_{j}^{+}\right)}^{2}}\;$$

where

(12) $$d_{BS}\left({\bar{S}}_{ij},S_{j}^{+}\right)={\left(\frac{1}{2}\left({\bar{S}}_{ij}-S_{j}^{+}\right)\tilde{S}{\left({\bar{S}}_{ij}-S_{j}^{+}\right)}^{T}\right)}^{\frac{1}{2}}.$$

The separation measure from NIBS, $D_{i}^{-}$ is given by:

(13) $${D_{i}^{-}=D}_{i}^{-}\left(O_{i},A^{-}\right)=\sqrt{\sum_{j=1}^{n}{w_{j}d_{BS}\left({\bar{S}}_{ij},S_{j}^{-}\right)}^{2}}$$

where

(14) $$d_{BS}\left({\bar{S}}_{ij},S_{j}^{-}\right)={\left(\frac{1}{2}\left({\bar{S}}_{ij}-S_{j}^{-}\right)\tilde{S}{\left({\bar{S}}_{ij}-S_{j}^{-}\right)}^{T}\right)}^{\frac{1}{2}}.$$

This quantifies the relative position of each alternative in the belief space.

Step 7. Calculating the Relative Closeness

The performance of each alternative is expressed by its relative closeness to the ideal solution:

(15) $${BT}_{i}=\frac{D_{i}^{-}}{D_{i}^{-}+D_{i}^{+}}$$

where $D_{i}^{-}$ and $D_{i}^{+}\;$ denote the distances of alternative $O_{i}$ from the negative and positive ideal belief solutions, respectively.

Step 8. Ranking and classifying the alternatives

Objects are ranked according to ${BT}_{i}\;$ values, where higher values indicate greater satisfaction. Based on the ordinal scale from $H_{1}\;$ to $H_{N}$, objects may also be classified by comparing their ratings with limiting profiles defined as: $S_{L1}$ = [0, 1, …, 0], $S_{LN-2}$ = [0, …, 1, 0]. Each object is assigned to the category of the closest profile, which can then be labelled using the corresponding linguistic term.

Step 9. Conducting the Sensitivity Analysis

The initial utility vector $U=\left[U\left(H_{1}\right),\ldots ,U\left(H_{N}\right)\right]$ is subjected to sensitivity analysis to reduce the dependence of outcomes on its subjective choice. Intermediate grades are allowed to vary within a specified range $U^{\prime }\left(H_{k}\right)\in [U\left(H_{k}\right)-\delta ,\;U\left(H_{k}\right)+\delta ]$. B-TOPSIS steps 3–7 are then repeated over R iterations using these perturbed utilities, generating sets of relative closeness values $BT_{i}={\left\{{BT}_{i}^{r}\right\}}_{r=1,\ldots ,R}$ for each object $O_{i}$. Objects are finally compared via stochastic analysis, e.g., significance tests or stochastic dominance applied to the distributions defined by $BT_{i}$ (see also [17]).

Optionally, sensitivity analysis can also be applied to criterion weights to examine the effect of weight variations on the final ranking.

**Figure 1:**
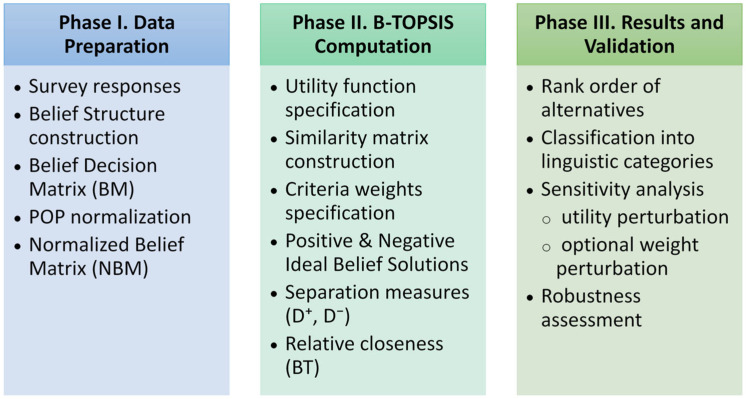
Overview of the B-TOPSIS Method for Aggregating and Ranking Ordinal Survey Responses.

The diagram (Figure 1) illustrates the key steps of the B-TOPSIS method, outlining the entire process from data encoding to final ranking.

## 4. Results

### 4.1. Cross-National and Socio-Demographic Patterns of Attitudes Toward Workplace AI

Section 4.1 addresses RQ1 by analysing EU27-level patterns, cross-country variation, and differences across demographic and occupational groups. Country-level results are based on Table A1, Table A2, Table A3, Table A4, Table A5, Table A6, Table A7 and Table A8 (Appendix A), while demographic and occupational breakdowns are reported in Table A9 (Appendix B). The interpretation follows Special Eurobarometer 554 [12], focusing on total positive responses.

#### 4.1.1. Statistical Analysis of Individual Questions

Across the EU27, attitudes toward AI in workplace functions vary systematically depending on the level of decision authority delegated to algorithms. When AI is used to gather additional information on job applicants (Q1), public opinion is moderate negative (43% positive, 50% negative). Support is higher in countries such as Sweden and Malta, while France shows consistently lower acceptance. Younger respondents, managers, white-collar workers, and individuals already aware of AI use in their workplace are more favourable, suggesting that both familiarity and occupational proximity to digital systems shape perceptions.

When AI moves from informational support to decision-making—such as selecting applicants for a job (Q2)—acceptance declines to 36% positive and 57% negative. This reduction is consistent across countries and highlights a clear sensitivity to the substitution of human judgment. Although Malta, Cyprus, and Sweden still show relatively higher support, France remains strongly sceptical. The same socio-demographic pattern persists, with younger individuals, managers, and AI-aware respondents more open to such applications, but overall attitudes become more cautious.

A different pattern emerges for task allocation and work scheduling (Q3), where attitudes are more balanced (49% positive, 44% negative). In this case, AI is more often perceived as an efficiency and coordination tool rather than a replacement for human authority. Acceptance is particularly high in Malta, Finland, and the Netherlands, while France again shows lower support. Managers, white-collar workers, and AI-aware respondents remain the most positive, reinforcing the idea that perceived augmentation rather than substitution increases acceptance.

Similarly balanced attitudes, yet with reversed fractions, are observed for collecting, processing, and storing employees’ personal data (Q4) (44% positive, 49% negative). While countries such as Malta and the Netherlands show relatively high acceptance, France and Greece express strong reservations. Occupational differences are evident, with managers and white-collar workers more supportive than manual workers. Across groups, awareness of AI use is associated with higher acceptance, although privacy concerns remain central.

A clear shift occurs when AI is applied to workplace safety and security (Q5), where acceptance is the highest among all items (67% positive, 25% negative). All demographic groups express majority support, particularly in Nordic countries. Even among less digitally engaged respondents, attitudes remain largely positive, suggesting that when AI is framed as protective rather than controlling, legitimacy increases substantially.

In contrast, monitoring workers (Q6) generates strong resistance across the EU27 (31% positive, 63% negative). This pattern is consistent across countries, with especially high rejection in Sweden, Germany, and Denmark. Even among respondents familiar with AI, acceptance remains limited, indicating strong sensitivity to perceived surveillance and loss of autonomy.

A similar but slightly less extreme pattern is observed for assessing workers’ performance (Q7) (36% positive, 57% negative). Nordic and Western European countries remain the most sceptical, while younger respondents and managers are somewhat more supportive. However, concerns about fairness, transparency, and control dominate across all groups.

The strongest rejection is observed for automatically firing workers (Q8), with only 16% positive and 78% negative responses. This pattern is nearly universal across countries and reflects deeply embedded norms regarding employment protection and human oversight. Even younger respondents and managers show only marginally higher acceptance.

#### 4.1.2. Comparative Insights: Country, Demographic, and Occupational Differences in AI Acceptance

Taken together, the results confirm strong and systematic differences in attitudes toward workplace AI across applications, countries, and socio-demographic groups. Overall acceptance ranges widely (16–67%), depending primarily on whether AI is perceived as augmenting human work or replacing human judgment.

Country-level patterns show a clear institutional dimension. Nordic countries combine high AI acceptance for efficiency-enhancing and safety-oriented applications with strong rejection of monitoring, performance evaluation, and automated dismissals. This reflects high-trust governance environments where AI is acceptable when it supports work but not when it constrains autonomy [18,27]. By contrast, France and several Central European countries exhibit more general scepticism, particularly regarding recruitment, data handling, surveillance, and dismissal decisions, reflecting stronger concerns about privacy and labour protection [6,7].

Demographic differences are also consistent across all items. Younger respondents show systematically higher AI acceptance across all workplace applications, especially in recruitment, task allocation, and safety-related uses. Older respondents are more cautious, particularly regarding monitoring and automated decisions. Gender differences are modest, with men slightly more positive overall, while women express greater concern regarding fairness and privacy. These results are consistent with prior research highlighting systematic demographic variation in attitudes toward AI in the workplace [50,51].

Occupational status introduces a clear gradient. Managers, self-employed individuals, and white-collar workers consistently report higher AI acceptance, particularly for efficiency-enhancing applications such as recruitment support and task allocation. In contrast, manual workers, unemployed respondents, and those in less secure positions show lower acceptance, especially for monitoring, performance evaluation, and automated dismissal, reflecting concerns about surveillance and job security. These findings are consistent with prior research showing that AI adoption depends on skills and adaptability, which are more prevalent among higher-status workers [52].

Finally, awareness and experience with AI emerge as one of the most consistent predictors of acceptance. Respondents aware of AI use in their workplace report significantly higher support across all applications compared to those without such awareness. This effect is particularly strong for recruitment and task allocation, suggesting that familiarity reduces perceived uncertainty and increases perceived legitimacy of AI systems [19,26].

Overall, these findings support RQ1 by showing that attitudes toward workplace AI are not uniform but structured by application type, institutional context, occupational position, and individual exposure. AI is most accepted when it is perceived as augmentative and least accepted when it is associated with surveillance, evaluation, or irreversible decisions affecting employment.

### 4.2. Application of the B-TOPSIS Framework to EU27 Countries

Section 4.2 addresses RQ2 and RQ3 by applying the B-TOPSIS method to survey data. The procedure generates both item-level rankings and an aggregated index, enabling comparative assessment of national acceptance of workplace AI. While descriptive measures facilitate comparison with official Eurobarometer reporting, they do not capture response intensity and uncertainty—limitations that B-TOPSIS explicitly addresses.

#### 4.2.1. B-TOPSIS Calculation for EU27 Countries

This section applies the B-TOPSIS framework to rank EU27 countries based on public attitudes toward workplace AI (Eurobarometer 554 [37]). Indicators BTQ1–BTQ8 correspond to eight AI workplace applications, while an aggregated BT index summarizes overall acceptance. Detailed formulas and the conceptual framework are presented in Section 3.2 (Figure 1).

Step 1. Respondents evaluated each AI application using a five-point scale: very positive, somewhat positive, somewhat negative, very negative, and don’t know. For the B-TOPSIS procedure, the four evaluative categories were encoded as belief levels $H_{1}-H_{4}\;$ (from very positive to very negative), while “don’t know” responses were treated as uncertainty. Raw distributions for all items are reported in Appendix A (Table A1, Table A2, Table A3, Table A4, Table A5, Table A6, Table A7 and Table A8).

Step 2. For each country and each item, response shares were transformed into belief structures. For illustration, responses from Belgium for Q1 are represented through Formula (1) as:

$$BS_{Q1}\left(BE\right)=\left\{{(H}_{1},0.06\right),(H_{2},0.44),(H_{3},0.32),(H_{4},0.15)\},\;\mathrm{with}\;\mathrm{uncertainty}\;=\;0.03.$$

This is equivalently expressed as a belief vector:

Q1(BE) = [0.06, 0.44, 0.32, 0.15].

Step 3. All belief vectors were normalised (Formula (4)) to ensure comparability across countries. For Belgium and Q1, we obtain:

NQ1(BE) = [0.062, 0.454, 0.330, 0.155].

Step 4. Each belief level was assigned a utility value [16,41]:

$$U\left(H_{1}\right)=1,\;U\left(H_{2}\right)=0.7,\;U\left(H_{3}\right)=0.4,U\left(H_{4}\right)=0.$$

The adopted utility values follow previous studies [15,16,41] employing belief-structure-based decision models. While alternative utility specifications are possible, the selected values preserve the ordinal interpretation of the response scale, providing a clear distinction between positive and negative evaluations, and facilitating comparison with prior research.

Based on these utilities, a similarity matrix was constructed to capture graded preferences (Formulas (6) and (7)) as follows:

$$\tilde{S}=\left[\begin{array}{cccc} 1 & 0.7 & 0.4 & 0\\ 0.7 & 1 & 0.7 & 0.3\\ 0.4 & 0.7 & 1 & 0.6\\ 0 & 0.3 & 0.6 & 1 \end{array}\right].$$

Step 5. For aggregated BT, all criteria were assigned equal weights, following standard practice in multi-criteria analysis when no empirical prioritisation is available [47,48,49]. This ensures balanced contribution of all workplace AI dimensions.

Step 6. Two reference solutions were defined. The Positive Ideal Belief Solution (PIBS) represents full acceptance of AI:

PIBS = [1, 0, 0, 0],

while the Negative Ideal Belief Solution (NIBS) represents complete rejection:

NIBS = [0, 0, 0, 1].

Step 7. Using belief-based distance measures (Formulas (11)–(14)), distances from PIBS and NIBS were computed. For Belgium and Q1:

$$D^{+}=0.5865,\;\mathrm{and}\;D^{-}=0.6056.$$

Step 8. Relative closeness coefficients were then calculated using the standard TOPSIS formulation (Formula (15)). For Belgium and Q1:

BTQ1=0.508.

The same procedure was applied to all eight indicators (BTQ1–BTQ8) and all EU27 countries. An aggregated BT index was also computed to summarize overall workplace AI acceptance. Equal weights were adopted in BT due to the absence of theoretical, empirical, or expert-based evidence supporting the differential importance of the analysed AI workplace applications. This approach is frequently employed in the MCDM literature when objective information regarding criterion importance is unavailable [47,48,49].

Finally, limiting profiles were defined to support comparative interpretation and clustering. The threshold values of 0.4495 and 0.6044 were derived from the B-TOPSIS evaluation of predefined limiting belief profiles, namely $S_{L1}$ = [0, 1, 0, 0], and $S_{L2}$ = [0, 0, 1, 0], representing adjacent acceptance categories. Based on these cut-off points, countries were classified into low (B-TOPSIS < 0.4495), medium [0.4495–0.6044], and high (B-TOPSIS > 0.6044) acceptance groups. This categorization provides a concise and methodologically grounded summary of cross-national differences in public acceptance of AI in the workplace.

The resulting closeness scores BTQ1–BTQ8, BT, and corresponding country rankings are reported in Table 2 and Table 3, respectively. Higher B-TOPSIS values indicate more favorable public attitudes toward AI.

Step 9. To capture uncertainty in the scoring function BT, we performed a Monte Carlo simulation with 5000 replications aimed at verifying the countries’ scores depending on the varying utility function. We allowed the utility values for the intermediate grades $H_{2}\;$ and $H_{3}$ to vary by ±0.1, i.e., $U(H_{2})\in \langle 0.6;0.8\rangle$ and $U(H_{3})\in \langle 0.3;0.5\rangle$. This approach accounted for possible differences in how respondents could interpret the linguistic scale.

The perturbation interval of ±0.1 was selected as a reasonable range around the baseline utility values, representing plausible variations in the perception of adjacent linguistic categories while preserving the ordinal structure of the utility function and avoiding category reversals. However, the exact magnitude of the perturbation can be adjusted depending on the specific context, data characteristics, and research objectives, and may be calibrated differently in alternative applications or by other researchers.

From the simulation, we obtain the sets $R_{i}$. The average simulated scores were the same as those obtained from step 8 for the initial U function, e.g., for Belgium, it equals 0.485. However, from the simulation, we were able to capture the variability of scores, e.g., for Belgium, the standard deviation of the BT value was 0.019, while the quantiles Q05–Q95 range was equal to 〈0.430; 0.495〉. In general, standard deviations were not high, they varied from 3.5% to 4.6% of the average BT values.

The average BT scores, calculated from the corresponding $R_{i}$ together with their standard deviations, are presented in Table 2, and the rank ordering in Table 3.

Finally, we verified whether the variability of $BT_{i}$ scores captured by the simulation allow the rank order of alternatives—derived as average from $BT_{i}^{r}$ values—to be confirmed. To this end, we conducted 351 pairwise one-sided Wilcoxon signed-rank tests with the alternative hypothesis that the median of paired differences in scores favours the higher-ranked alternative. Since all tests were performed simultaneously, p-values were adjusted using the Holm–Bonferroni correction. The results show that for all pairs, the BT scores of higher-ranked countries are significantly greater than those of lower-ranked countries (*p* < 0.01).

**Figure 2:**
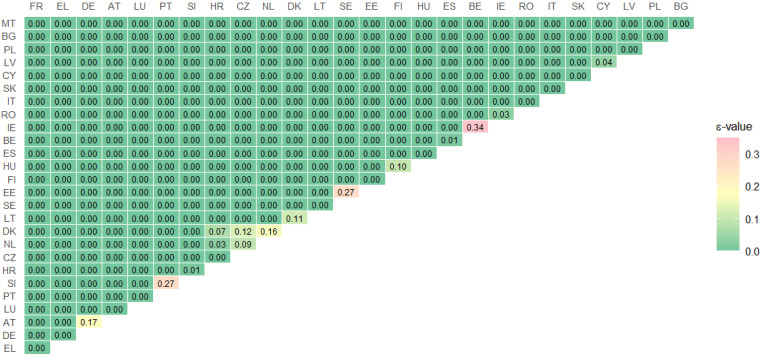
The heatmap of ε values in ASD-based analysis of $BT_{i}$-base distribution values of countries.

To verify whether these results hold regardless of the decision maker’s attitude toward the risk of misclassifying countries, we performed a stochastic dominance analysis based on Almost Stochastic Dominance (ASD) [53]. The heatmap of ε domination violation areas in ASD is shown in Figure 2.

In Figure 2, each row represents a dominating country and each column a dominated one. The ε values indicate the extent of dominance violation, defined as the fraction of the total area between the two empirical CDFs over which the supposedly dominated distribution lies above the dominating one. The majority of cells show ε=0.00, indicating that First-order Stochastic Dominance (FSD) holds for most pairwise comparisons. In cases where FSD is violated, ε values remain well below the limiting $\varepsilon^{*}=0.5$ threshold, confirming that ASD holds.

However, some researchers suggest more restrictive values of $\varepsilon^{*}$ as benchmarks, as experimentally verified and reflecting the proportion of rational decision makers whose preferences would be consistent with the dominance relation [54]. Following this reasoning, we adopt $\varepsilon^{*}=0.2$ as a limiting threshold for confirming ASD. Three pairs in our analysis exceed this threshold: IE–BE (ε=0.34), EE–SE (ε=0.27), and SI–PT (ε=0.27). Countries within these pairs are therefore considered non-dominant with respect to $BT_{i}$ scores and are assigned equal rank positions: IE and BE share 9th place, EE and SE share 14th place, and SI and PT share 21st place.

Overall, accounting for the variability induced by changes in the shape of the utility function alters the final ranking only marginally, with a small number of country pairs treated as tied rather than strictly ordered.

#### 4.2.2. Individual B-TOPSIS Indexes of Workplace AI Acceptance

The analysis of B-TOPSIS variables across the EU27 reveals both common tendencies and marked cross-country divergences, providing a clear answer to RQ2: How do EU Member States rank with respect to individual AI-related workplace activities when evaluated using the B-TOPSIS method?

**Figure 3:**
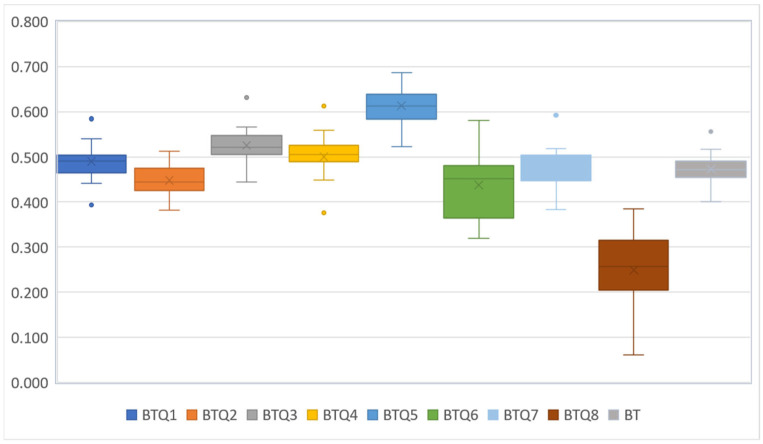
Box plots illustrating the distribution of values for BTQ1–BTQ8 and BTQ.

Figure 3 presents box plots for BTQ1–BTQ8 and BT, illustrating distributional characteristics including median, dispersion, and outliers.

Across the eight indicators, mean values range from approximately 0.249 for automatic dismissal (BTQ8) to above 0.614 for improving workers’ safety and security (BTQ5), indicating substantial variation in perceived acceptability across workplace AI applications (Figure 3). Dispersion is particularly high for ethically sensitive and control-oriented uses. Automatic dismissal (BTQ8) shows the widest spread, ranging from 0.060 in Sweden to 0.385 in Poland. High variability is also observed for employee monitoring (BTQ6), ranging from 0.319 in Austria to 0.580 in Malta. By contrast, recruitment-related practices (BTQ2) and safety-oriented applications (BTQ5) display lower dispersion, reflecting more homogeneous acceptance across Member States. Overall, surveillance and sanction-related applications generate substantially greater cross-national divergence than efficiency- or safety-oriented uses.

Descriptive statistics and outlier patterns further confirm these differences (Table 2, Figure 3). A small group of countries consistently occupies the upper range across multiple indicators, particularly for operational and safety-related applications. In contrast, other countries remain persistently at the lower end, especially for recruitment and more intrusive applications. For instance, France records the lowest values for gathering applicant information (BTQ1 = 0.394) and processing employee data (BTQ4 = 0.376), while Sweden and Denmark show the lowest acceptance for automatic dismissal (BTQ8 = 0.060 and 0.090, respectively).

Applying the B-TOPSIS classification thresholds—low (0–0.449), medium (0.449–0.604), and high (0.604–1]—provides a clearer interpretation. Most EU Member States fall into the medium acceptance category, indicating generally cautious but open attitudes toward workplace AI. Medium acceptance dominates recruitment (BTQ1), operational tasks (BTQ3–BTQ4), and managerial functions such as monitoring and performance evaluation (BTQ6–BTQ7). High acceptance is concentrated in safety-related applications (BTQ5), whereas automatic dismissal (BTQ8) is uniformly classified as low acceptance across all countries.

Overall, EU Member States systematically distinguish between types of AI workplace applications: recruitment and operational uses are mostly moderately accepted, safety-related applications are broadly supported, and ethically sensitive uses—particularly monitoring and automated dismissal—are strongly rejected.

#### 4.2.3. Aggregated B-TOPSIS Index of Workplace AI Acceptance

The aggregated B-TOPSIS index provides a synthetic overview of EU Member States’ attitudes toward AI-driven HR practices, addressing RQ3: How do EU Member States rank on an aggregated B-TOPSIS index capturing multidimensional attitudes toward workplace AI?

Aggregated BT scores range from 0.401 in France to 0.556 in Malta. Countries that score highly across recruitment, operational, and monitoring dimensions—such as Malta, Bulgaria, Cyprus, Latvia, and Poland—also rank at the top of the aggregated index (Table 2). Malta emerges as the overall leader, reflecting consistently high acceptance across most AI applications. At the lower end, France (0.401), Greece (0.435), Germany (0.441), and Austria (0.443) exhibit the most cautious attitudes, consistent with their low acceptance of intrusive or ethically sensitive applications in the disaggregated results. Intermediate positions are occupied by countries such as Italy, Ireland, and Poland (0.491–0.507), indicating moderate and context-dependent acceptance.

Applying the same thresholds to the aggregated index shows a more compressed distribution compared to individual indicators. Most Member States (22 of 27) fall into the medium acceptance category. Only France, Greece, Germany, Austria, and Luxembourg are classified as low acceptance, while no country reaches the high acceptance category at the aggregate level. This reflects a smoothing effect: extreme differences observed in individual applications—especially monitoring (BTQ6) and automatic dismissal (BTQ8)—are reduced when combined into a single index, although relative country rankings remain largely stable.

**Figure 4:**
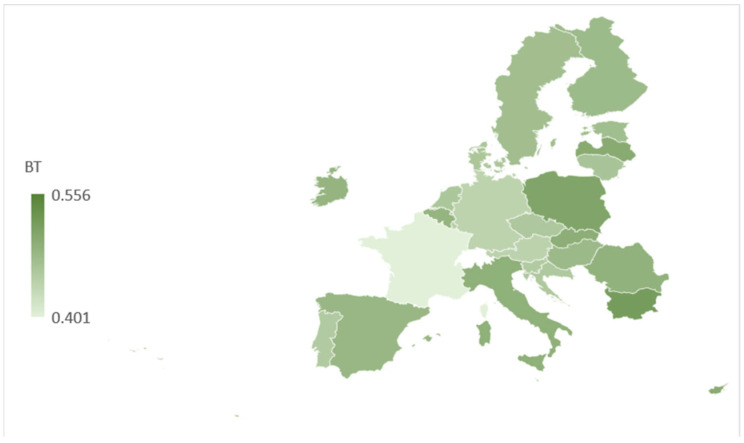
Workplace AI acceptance across EU countries based on BT values.

Figure 4 illustrates cross-country variation in aggregated workplace AI acceptance.

Countries that consistently perform well in individual indicators—such as Malta, Finland, Denmark, and Latvia—also rank highly in the aggregated index, while more cautious countries such as France, Sweden, Germany, and Austria remain at the lower end. Although aggregation reduces dispersion and masks some within-country variation (particularly mixed attitudes across different AI uses), the overall structure of differences remains stable.

In sum, the aggregated ranking confirms the patterns observed in individual indicators: workplace AI acceptance in Europe is heterogeneous, with a clear division between high-acceptance, intermediate, and low-acceptance countries. High-ranking countries show consistently positive attitudes across most workplace functions, while low-ranking countries remain cautious, particularly regarding ethically sensitive applications. Intermediate countries adopt a selective approach, supporting certain uses while remaining critical of others.

## 5. Discussion

### 5.1. Comparison of Rankings Based on Total Positive Responses and B-TOPSIS for the Individual Questions

To evaluate cross-country attitudes toward AI in the workplace, we compare country rankings based on “Total positive” responses, defined as the combined share of “Very positively” and “Somewhat positively” responses (Table A1, Table A2, Table A3, Table A4, Table A5, Table A6, Table A7 and Table A8 in Appendix A), with those derived from the B-TOPSIS method (BTQ1–BTQ8). The analysis is performed separately for each survey item. Unlike the “Total positive” measure, which focuses solely on the overall proportion of positive responses, B-TOPSIS takes into account the full distribution of responses, including their intensity, negative evaluations, and uncertainty.

Across all eight workplace AI applications, the two approaches produce highly correlated results. Pearson correlations between the conventional total positive-response measure (Table A1, Table A2, Table A3, Table A4, Table A5, Table A6, Table A7 and Table A8 in Appendix A) and the corresponding B-TOPSIS scores (Table 2) range from 0.908 (Q5) to 0.972 (Q6), while Spearman rank correlations range from 0.854 (Q5) to 0.964 (Q6) (Table 4).

These values indicate substantial overall consistency between the two approaches. At the same time, the correlations are not perfect, suggesting that meaningful differences emerge once the full belief structure is taken into account. Importantly, high correlation should not be interpreted as methodological equivalence. Correlation coefficients capture overall association but do not reveal differences in the relative positions of individual countries. Consequently, even highly correlated rankings may contain substantively important shifts that influence comparative interpretation. Notably, the lowest rank correlation is observed for Question Q5 (improving workers’ safety and security), suggesting that response intensity, negativity, and uncertainty exert a stronger influence on the resulting rankings in this domain. In contrast, Questions Q6 (monitoring workers) and Q8 (automatically firing workers), which are characterized by more polarized attitudes, exhibit stronger agreement between the two approaches.

To illustrate these patterns in more detail, several individual questions are discussed below. Question Q1 (gathering additional information on applicants for a job) provides a useful example. Although the correlation between the percentage of positive responses and BTQ1 is high (Pearson = 0.964; Spearman = 0.939), the two rankings are not identical. B-TOPSIS differentiates between countries that appear identical under conventional aggregation. For example, Belgium, Bulgaria, and Hungary each report 50% positive responses and are therefore tied under the simple measure. However, BTQ1 distinguishes between them, assigning values of 0.540 for Bulgaria, 0.508 for Belgium, and 0.501 for Hungary, with Bulgaria ranking highest. This reflects differences in the full response structure, including Bulgaria’s higher share of “very positive” responses (10%), lower negativity (33%), and moderate uncertainty (17%), compared with Belgium’s higher negativity (47%) and low uncertainty (3%), and Hungary’s more balanced distribution of responses (50% positive and 45% negative) (Appendix A: Table A1). By incorporating the full belief distribution, B-TOPSIS distinguishes countries that appear identical under simple positive-response aggregation.

A similar pattern is observed in Question Q2 (selecting applicants for a job). Hungary and Poland both report 41% positive responses, yet Poland achieves a higher B-TOPSIS score (0.472 versus 0.447), primarily due to Hungary’s larger proportion of strongly negative responses (29% compared with 20% in Poland). Thus, while conventional aggregation treats the countries as equally supportive, B-TOPSIS identifies a substantially more favourable overall attitudinal profile in Poland.

In Question Q4 (collecting, processing, and storing workers’ personal data), Bulgaria and Italy both report 55% positive responses. However, Bulgaria achieves a higher B-TOPSIS score (0.558 versus 0.527), as opposition to AI-supported data processing is considerably lower in Bulgaria (30% compared with 41% in Italy). Likewise, in Question Q5 (improving workers’ safety and security), Belgium and Germany both record 70% positive responses, yet Germany obtains a higher B-TOPSIS score (0.620 compared with 0.595), despite identical levels of support, due to lower negative sentiment (21% versus 28%).

Conversely, countries with different levels of positive responses may exhibit nearly identical belief structures.

For example, Romania reports 46% positive responses, whereas Hungary reaches 50%, yet both countries obtain almost the same BTQ1 value (0.500 for Romania and 0.501 for Hungary). This reflects similar overall attitudinal balances, characterized by comparable proportions of negative responses (44% in Romania and 45% in Hungary) and moderate levels of uncertainty (10% and 5%, respectively). While the simple percentage measure highlights a four-point difference in positivity, B-TOPSIS captures that the underlying belief structures—and thus the relative distances from the ideal and anti-ideal solutions—are almost identical. In highly polarized cases, both methods converge; for example, Sweden and Malta consistently rank highly under both approaches due to strong positive sentiment and low uncertainty.

Finally, in Question Q8 (automatically firing workers), Poland and Romania illustrate another case of near convergence. Although Poland records 33% positive responses compared with 28% in Romania, their B-TOPSIS scores are almost identical (0.385 and 0.383, respectively). This results from compensating differences in the distribution of negative and uncertain responses, which offset differences in positivity and lead to similar positions relative to the ideal and anti-ideal solutions.

A detailed comparison between Total Positive Responses and the B-TOPSIS framework is provided in [15], where both approaches are systematically contrasted across several methodological dimensions, including response range, opinion intensity, treatment of missing data, accuracy of insight, computational complexity, use case, potential drawbacks, advantages, and main recommendations. In the present study, Table 5 summarises the key differences between the two approaches, focusing on the most relevant aspects for our analysis of survey-based evaluation methods. This provides a simplified overview of the main conceptual distinctions relevant for interpreting the results.

Overall, the practical value of B-TOPSIS lies not in producing radically different rankings, but in preserving information that is not captured by conventional positive-response measures. While both approaches identify similar broad patterns of acceptance and resistance toward workplace AI, B-TOPSIS additionally accounts for response intensity, opposition, and uncertainty, enabling a more nuanced interpretation of cross-country differences, particularly when countries exhibit similar levels of overall support but different underlying response structures.

### 5.2. Individual Versus Total B-TOPSIS Measure

Table 6 summarizes rankings across BTQ1–BTQ8 and the aggregated BT index, showing top and bottom countries and acceptance classes.

The relationships between BTQ1–BTQ8 and BT variables were assessed using Pearson’s correlation coefficient, with results summarized in Table 7.

Correlation analysis (Table 7) reveals clear clustering patterns. Recruitment variables (BTQ1–BTQ2) are highly correlated (0.902), indicating that countries perceive early stages of the hiring process in a similar way.

AI-supported workforce management activities, including task allocation, employee data handling, monitoring, and performance evaluation (BTQ3, BTQ4, BTQ6, BTQ7), exhibit moderate to strong correlations (0.424–0.895), except for BTQ3 and BTQ6, which show a weak relationship (0.263).

Within this group, monitoring (BTQ6) and performance assessment (BTQ7) are particularly strongly linked (0.895), suggesting that they form a coherent cluster of algorithmic management practices perceived as part of integrated managerial oversight.

In contrast, safety-oriented AI (BTQ5) and automatic dismissal (BTQ8) are weakly or negatively correlated with most other variables, with a strong negative relationship between them (−0.734). This confirms that safety-related AI is perceived as a distinct, non-surveillance dimension, whereas automatic dismissal represents a separate and ethically sensitive category.

The aggregated B-TOPSIS index further clarifies these patterns by smoothing extreme values observed in individual variables, particularly for monitoring and automatic dismissal, while preserving overall country rankings. Most EU countries (22 of 27) fall into the medium acceptance class, five remain in the low class, and none achieve uniformly high acceptance across all dimensions. This confirms a generally cautious but not rejectionist stance toward workplace AI across the EU.

Beyond individual rankings and threshold-based classifications, the results reveal recurring patterns in how countries combine acceptance levels across different AI applications. The profiles discussed below are illustrative, highlighting analytically meaningful configurations rather than formally derived clusters.

At the high end of the spectrum, Malta (BT 1—first position) exemplifies broad acceptance across multiple domains. It reaches the high acceptance class for task allocation (BTQ3), processing employee data (BTQ4), and workplace safety (BTQ5), while maintaining medium acceptance for recruitment-related practices (BTQ1–BTQ2) and employee monitoring (BTQ6). Even in this leading position, automatic dismissal (BTQ8) remains in the low acceptance class, underscoring its universal ethical sensitivity.

At the opposite extreme, France (BT 27—last position) exhibits a consistently low-acceptance profile across most AI applications. It falls into the low acceptance class for recruitment-related data practices (BTQ1, BTQ4), monitoring (BTQ6), performance evaluation (BTQ7), and automatic dismissal (BTQ8), while safety-oriented AI (BTQ5) reaches only the medium class. This indicates persistent caution toward AI-enabled managerial and control-oriented applications.

Several intermediate configurations emerge. Bulgaria (BT 2), Latvia (BT 4), Cyprus (BT 5), Slovakia (BT 6), and Ireland (BT 9) combine high acceptance of safety-oriented AI (BTQ5) with medium acceptance across recruitment, operational, and monitoring-related applications (BTQ1–BTQ4, BTQ6–BTQ7). These countries appear receptive to AI as a tool for efficiency and protection, while maintaining reservations toward automated dismissal (BTQ8).

Poland (BT 3), Italy (BT 7), and Romania (BT 8) display relatively stable profiles, with medium acceptance across most applications (BTQ1–BTQ7) and low acceptance for automatic dismissal (BTQ8), aligning with the dominant EU-wide pattern.

A distinct Nordic profile is observed in Sweden (BT 15) and Denmark (BT 17). Both countries combine very high acceptance of safety-oriented AI (BTQ5) with low acceptance of monitoring (BTQ6), performance evaluation (BTQ7), and especially automatic dismissal (BTQ8). Recruitment and operational applications (BTQ1–BTQ4) remain in the medium acceptance class, reflecting a clear normative separation between AI that supports worker well-being and AI associated with surveillance or disciplinary control.

Luxembourg (BT 23) and Greece (BT 26) show a similarly selective pattern. Both exhibit very high acceptance of safety-related AI (BTQ5), while maintaining low acceptance for recruitment (BTQ2), operational processing (BTQ4), monitoring (BTQ6), and automatic dismissal (BTQ8). Performance evaluation (BTQ7) remains in the medium class. This reflects a cautious, risk-sensitive approach to AI adoption, prioritising ethically uncontroversial applications.

Finally, the Netherlands (BT 18), Austria (BT 24), and Germany (BT 25) also display selective profiles: medium acceptance for recruitment and operational uses (BTQ1, BTQ3, BTQ4), high acceptance for safety-oriented AI (BTQ5), and low acceptance for monitoring, performance evaluation, and automatic dismissal (BTQ6–BTQ8). This indicates openness to efficiency and safety gains combined with strong reservations toward algorithmic control mechanisms.

Taken together, the results synthesize evidence from both individual B-TOPSIS variables and the aggregated index, revealing stable and structured cross-national differences in workplace AI acceptance. The aggregated measure does not obscure these differences; instead, it clarifies them by reducing noise while preserving meaningful variation in country-level profiles and their underlying ethical and functional boundaries.

### 5.3. Sensitivity Analysis of BT Measure

The use of Monte Carlo simulation in the B-TOPSIS procedure enhances the reliability of the results by accounting for uncertainty in model parameters, particularly those that must be specified arbitrarily, such as utility values. By repeating the calculations under multiple plausible parameter scenarios, it is possible to test the stability of the obtained scores and identify alternatives that remain robust despite small variations in assumptions. Furthermore, the application of ASD allows for a more flexible comparison of alternatives than strict stochastic dominance, reducing the number of incomparable pairs and leading to a more informative final ranking.

**Figure 5:**
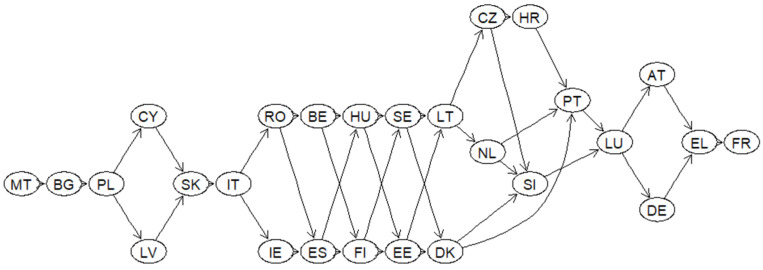
Hasse diagram reflecting FSD-based dominance relationships derived from simulated $BT_{i}$ score distributions.

It is worth noting that if pure FSD were applied, beyond the three pairs of countries considered equivalent under ASD (IE and BE, EE and SE, and SI and PT), several additional pairs would also fail to dominate one another. Examining the non-zero ε values in Figure 3, we find that HU and FI, LT and DK, DK and HR, DK and CZ, DK and NL, NL and HR, NL and CZ, and AT and DE should all be treated as mutually incomparable. Consequently, when FSD is applied, the ranking of countries is considerably less resolved—for instance, while LT and DK would be considered equivalent, and similarly, DK and NL, LT nonetheless dominates NL. A Hasse diagram based on top-down filtration can be used to represent confirmed dominance relations and facilitate interpretation by decision makers. Such a diagram is presented in Figure 5.

The choice between FSD and ASD remains an analytical decision that should reflect the decision makers’ preferences and their attitudes toward interpretive risk. In the present study, which aims to analyse AI acceptance across EU countries in as objective a manner as possible, FSD would be appropriate if one requires results to remain valid for even the most conservative interpreters—those who do not permit even the smallest risk of misclassification. Under such an approach, the results revealed by FSD and shown in Figure 5 should form the basis for interpretation, with the caveat that they entail numerous incomparabilities. However, if a minor risk of misclassification is acceptable—a tolerance empirically shown to be common among decision makers [54]—then ASD with the assumed threshold ε* provides a more decisive basis for interpretation. The resulting ranking is largely consistent with that obtained from a single B-TOPSIS analysis under an arbitrarily assumed utility function, with the exception of three pairs of countries identified as tied.

### 5.4. Key Findings and Their Interpretation

The empirical analysis provides several important insights into public attitudes toward workplace AI across the EU27. The findings demonstrate that acceptance of AI strongly depends on the specific workplace function performed by AI systems, as well as on national, demographic, and occupational characteristics.

First, the results confirm that European citizens clearly distinguish between supportive and control-oriented AI applications. AI systems designed to improve workers’ safety and security received the highest levels of acceptance across the EU27, indicating broad public support for technologies perceived as protective and efficiency-enhancing. In contrast, applications involving employee monitoring, performance evaluation, and especially automatic dismissal generated substantially lower acceptance and stronger ethical concerns.

Second, significant cross-country differences were identified. Malta, Bulgaria, Cyprus, Latvia, and Poland emerged as the countries with the highest overall acceptance of workplace AI, while France, Germany, Austria, Greece, and Luxembourg showed more sceptical attitudes. Nordic countries displayed a distinctive pattern characterized by high support for safety-oriented AI combined with strong rejection of surveillance and disciplinary applications. These findings suggest that institutional context, labour market culture, and social trust play an important role in shaping attitudes toward workplace AI.

Third, socio-demographic and occupational characteristics systematically influenced AI acceptance. Younger respondents, managers, white-collar workers, and individuals already familiar with AI technologies in their workplaces were consistently more supportive of AI applications. In contrast, older respondents, manual workers, and individuals in less secure employment positions expressed greater concerns, particularly regarding algorithmic monitoring and automated decision-making. This indicates that digital familiarity and labour market position significantly shape perceptions of AI legitimacy.

Fourth, the study demonstrates the methodological advantages of the B-TOPSIS framework compared with traditional percentage-based measures. By incorporating the full distribution of responses, including response intensity and uncertainty, B-TOPSIS provides a more comprehensive decision-making framework for assessing public attitudes, while preserving the informational richness of response distributions.

The method was able to differentiate countries with similar levels of positive responses but different underlying belief structures and uncertainty patterns.

Fifth, the robustness analysis confirmed the stability of the obtained rankings. Monte Carlo simulations and stochastic dominance analysis showed that the relative positions of countries remained largely unchanged under alternative utility assumptions, with only a limited number of country pairs requiring treatment as statistically equivalent. This confirms the reliability and decision robustness of the proposed analytical framework.

Six, the results indicate that public acceptance of workplace AI is not uniform but varies systematically across organisational functions. The most pronounced distinction concerns the contrast between applications supporting employees and those associated with managerial control. Across the EU27, AI systems aimed at improving workplace safety receive the highest levels of acceptance, whereas monitoring, performance evaluation, and especially automated dismissal are significantly less supported. This suggests that citizens do not evaluate workplace AI as a homogeneous technological category, but instead assess specific applications based on their perceived consequences for employees and organisations. This pattern suggests an implicit evaluative boundary between assistive and controlling applications of workplace AI.

These findings align with research on technology acceptance and trust in AI systems, which identifies perceived fairness, transparency, autonomy, and organisational trust as key determinants of attitudes toward AI technologies [55,56,57]. Although these studies are not specific to workplace management contexts, they point to general mechanisms of AI acceptance that appear transferable across domains. AI applications that augment human work and enhance safety are therefore likely perceived more positively, as they provide clear benefits while preserving human oversight, whereas systems used for surveillance or automated decision-making raise concerns regarding legitimacy, accountability, and the displacement of human judgment.

Additional insights come from recent AI acceptance studies, though mostly conducted in education [58,59,60] and service contexts [61] rather than workplace governance. Bai and Yang [60], using the extended AIDUA (Acceptance of Artificial Intelligence and Data Analytics) framework, emphasise that ethical risk perceptions and explainability are central to AI acceptance beyond performance expectations. While their empirical setting differs, the underlying cognitive and ethical mechanisms are consistent with the patterns observed in the present study. This helps explain why workplace safety applications are broadly supported, whereas automated dismissal—the most consequential and controversial application analysed—consistently receives low acceptance across Europe.

Similarly, Luo and Cao [59], show that trust, compatibility, and perceived usefulness significantly shape intentions to adopt AI. Although focused on generative AI rather than workplace systems, their findings suggest a broadly consistent role of trust and perceived benefit across application domains. In the workplace context, this implies that AI systems perceived as useful, understandable, and aligned with socially desirable organisational goals are more likely to be accepted than those associated with monitoring or disciplinary functions. This pattern is consistent with the strong support observed for safety-oriented applications across EU Member States.

The results also support the view that AI acceptance is multidimensional rather than reflecting a single pro- or anti-technology attitude. As argued by Jefmański et al. [61] acceptance should be analysed across multiple attitudinal dimensions rather than through a single global index. The present findings reinforce this claim by showing substantial variation across workplace functions—such as recruitment, operational management, safety, monitoring, performance evaluation, and dismissal—despite moderate values of the aggregated BT index.

Overall, the evidence suggests that workplace AI acceptance is shaped by a combination of perceived utility, trust, ethical considerations, and expectations regarding human oversight. Attitudes toward AI therefore depend less on the technology itself and more on the organisational functions it serves and the extent to which it is perceived as supporting rather than replacing human judgment.

Finally, from a regulatory perspective, the results indicate that acceptance varies meaningfully across functional domains, suggesting implicit societal boundaries for algorithmic decision-making in the workplace. These boundaries are closely related to the degree of human oversight retained in employment-related processes, with significantly lower acceptance for systems that substitute rather than support human judgment. This supports the need for functionally differentiated, risk-based regulatory approaches in workplace AI governance, consistent with the logic of the EU AI Act [8], particularly through clearer distinctions between assistive applications and high-impact decision-making systems in employment contexts.

## 6. Conclusions, Limitations, and Future Research

This study applied the Belief Structure TOPSIS (B-TOPSIS) method to analyse EU27 attitudes toward AI in the workplace. By preserving the ordinal structure of survey responses and explicitly incorporating uncertainty, the approach allowed retaining information that is typically lost in conventional percentage-based measures.

The results indicate that attitudes toward workplace AI are strongly context-dependent. AI applications supporting operational tasks and workplace safety receive relatively high acceptance across the EU, whereas monitoring, performance evaluation, and especially automatic dismissal remain considerably more controversial. Although most EU countries fall within a medium acceptance category, meaningful cross-country differences persist, reflecting variation in institutional environments, labour market cultures, and levels of trust in AI technologies.

A methodological contribution of the study is also important. Compared with traditional measures based solely on positive responses, B-TOPSIS additionally accounts for response intensity, negative evaluations, and uncertainty. The comparison of rankings shows that while both approaches identify similar broad patterns, meaningful differences emerge when the full response distribution is considered. Robustness analyses based on Monte Carlo simulation and stochastic dominance further confirm the stability of the obtained rankings.

At the same time, socio-demographic patterns indicate higher acceptance among younger, urban, and more AI-experienced respondents, while older and rural populations remain more cautious, particularly regarding monitoring and automated decision-making.

Despite these advantages, the study has several limitations. It relies on cross-sectional survey data and therefore does not capture temporal dynamics or actual behavioural adoption of AI systems. In addition, the use of equal weights may mask differences in the ethical and social importance of individual indicators.

However, the Eurobarometer data do not capture key psychological determinants of AI acceptance—such as trust, perceived usefulness, ethical risk, or explainability—which limits the ability to explain the observed differences across workplace AI applications. Future research based on richer survey instruments is therefore needed to move beyond descriptive patterns toward mechanism-based explanations.

Future research may extend the proposed framework in several directions. First, longitudinal analyses could be used to examine how attitudes toward workplace AI evolve over time. Second, alternative weighting schemes may be explored. While equal weights were adopted due to the absence of a clear theoretical or empirical basis for differential weighting, expert-based approaches such as AHP (Analytic Hierarchy Process) [62], FUCOM (Full Consistency Method) [63], BWM (Best Worst Method) [64], or SWARA (Step-wise Weight Assessment Ratio Analysis) [65], may provide more behaviourally grounded weighting structures.

Another direction concerns weighting procedures tailored to belief-structure-based decision frameworks. Since classical entropy methods are designed for crisp data, they cannot be directly applied to belief distributions without loss of information. Future research may therefore develop entropy-inspired measures adapted to belief structures while preserving uncertainty.

A potential direction for future research is the empirical calibration of utility values based on dedicated studies on the perception of linguistic scales, as well as a systematic comparison of alternative utility specifications to further assess the sensitivity of the proposed framework.

Methodologically, further work may integrate belief-structure representations with alternative reference-point MCDM methods. Beyond B-TOPSIS, this includes approaches such as Hellwig’s approach [66,67], MARCOS (Measurement of Alternatives and Ranking according to COmpromise Solution), EDAS (Evaluation based on Distance from Average Solution) [68], and other compromise-ranking techniques. Since VIKOR (VIšekriterijumsko KOmpromisno Rangiranje, i.e., Multi-Criteria Optimization and Compromise Solution) [69] has already been extended to uncertainty-oriented environments, similar adaptations for belief structures appear promising and may improve the stability of results across different decision logics.

Additionally, future studies may compare the obtained results with those derived from alternative MCDM approaches tailored to survey data analysis, including Intuitionistic Fuzzy TOPSIS, Intuitionistic Fuzzy Hellwig method, and Interval Intuitionistic Fuzzy TOPSIS, to evaluate the stability, reliability, and sensitivity of the resulting rankings. Future work may involve benchmarking the results against intuitionistic and interval intuitionistic fuzzy methods [70,71,72] specifically designed for survey data analysis.

Finally, future research may explore hybrid AI-assisted decision-support systems combining large language models (LLMs) [73,74] with MCDM methods, where LLMs support problem formulation and interpretation, while MCDM ensures transparent handling of uncertainty and preference aggregation.

In sum, the findings suggest that public acceptance of workplace AI depends not only on the technology itself but also on its perceived purpose and consequences for workers. While conventional survey indicators capture broad patterns of support, incorporating response intensity, opposition, and uncertainty provides a more nuanced understanding of public attitudes. Beyond this application, the proposed B-TOPSIS framework offers a flexible tool for analysing technology acceptance and other opinion phenomena under uncertainty.

## Figures and Tables

**Table 1:** Mapping Literature and Survey Evidence to AI-Enabled Workplace Functions.

AI-Enabled Workplace Function	Key References	Key Insights
Recruitment and Candidate Selection	[19,21,22,23,24]	AI improves recruitment efficiency, supports candidate evaluation, and may reduce bias. Acceptance depends on fairness, transparency, and human oversight.
Task Allocation and Workforce Management	[18,19,20,25,26,27,28,29]	AI can optimize workload distribution and scheduling, but its impact on autonomy depends on implementation and governance arrangements.
Data Collection, Processing, and Storage	[5,6,7,30,32]	AI strengthens data management capabilities but raises concerns related to privacy, bias, and ethical oversight.
Workplace Safety and Security	[20,25,30,31]	AI can improve workplace safety and risk detection, although concerns remain regarding employee autonomy and surveillance.
Employee Monitoring	[5,30,32]	Monitoring systems may improve efficiency and control, but often generate privacy concerns and reduce perceived trust.
Performance Assessment	[18,19,24,25,33,34]	AI can support faster and more consistent performance evaluation, but trust depends on transparency, fairness, and accountability.
Automated Managerial Decisions (including Dismissals)	[24,25,30,33,35,36]	Automated decisions in sensitive HR areas raise ethical, legal, and social concerns, particularly when human oversight is limited.

**Table 2:** Assessment of acceptance with AI in the workplace in European countries using BTQ1–BTQ8 and BT.

Country	BTQ1	BTQ2	BTQ3	BTQ4	BTQ5	BTQ6	BTQ7	BTQ8	BT	Av BT ($R_{i}$)	SD
EU27	0.475	0.443	0.508	0.483	0.607	0.42	0.452	0.266	0.462	0.462	0.019
Belgium (BE)	0.508	0.474	0.528	0.513	0.595	0.444	0.474	0.315	0.485	0.485	0.021
Bulgaria (BG)	0.54	0.506	0.556	0.558	0.639	0.51	0.509	0.3	0.518	0.518	0.021
Czechia (CZ)	0.468	0.428	0.492	0.492	0.597	0.434	0.46	0.257	0.458	0.458	0.020
Denmark (DK)	0.504	0.453	0.558	0.536	0.685	0.332	0.414	0.09	0.46	0.461	0.017
Germany (DE)	0.465	0.434	0.521	0.464	0.62	0.327	0.387	0.243	0.441	0.442	0.019
Estonia (EE)	0.492	0.444	0.558	0.489	0.615	0.406	0.51	0.193	0.472	0.472	0.019
Ireland (IE)	0.493	0.45	0.54	0.525	0.633	0.467	0.494	0.233	0.485	0.486	0.019
Greece (EL)	0.466	0.444	0.507	0.381	0.605	0.342	0.464	0.215	0.435	0.436	0.018
Spain (ES)	0.488	0.434	0.503	0.506	0.653	0.479	0.489	0.267	0.481	0.481	0.018
France (FR)	0.394	0.382	0.444	0.376	0.579	0.36	0.403	0.205	0.401	0.401	0.018
Croatia (HR)	0.471	0.428	0.481	0.489	0.566	0.424	0.457	0.321	0.457	0.457	0.020
Italy (IT)	0.497	0.48	0.517	0.527	0.583	0.481	0.486	0.337	0.491	0.491	0.021
Republic of Cyprus (CY)	0.526	0.484	0.524	0.511	0.613	0.472	0.518	0.313	0.498	0.498	0.020
Latvia (LV)	0.502	0.476	0.547	0.52	0.62	0.526	0.519	0.231	0.499	0.499	0.020
Lithuania (LT)	0.45	0.413	0.513	0.5	0.575	0.47	0.489	0.247	0.463	0.463	0.020
Luxembourg (LU)	0.46	0.418	0.493	0.448	0.622	0.414	0.455	0.208	0.448	0.448	0.018
Hungary (HU)	0.501	0.447	0.515	0.5	0.568	0.449	0.489	0.325	0.477	0.477	0.020
Malta (MT)	0.587	0.513	0.631	0.612	0.667	0.58	0.592	0.265	0.556	0.557	0.020
Netherlands (NL)	0.487	0.441	0.55	0.545	0.641	0.364	0.399	0.139	0.459	0.46	0.018
Austria (AT)	0.465	0.424	0.547	0.451	0.632	0.319	0.384	0.258	0.443	0.443	0.016
Poland (PL)	0.525	0.472	0.52	0.538	0.569	0.514	0.512	0.385	0.507	0.508	0.022
Portugal (PT)	0.473	0.425	0.473	0.472	0.589	0.452	0.447	0.271	0.454	0.454	0.019
Romania (RO)	0.5	0.47	0.508	0.495	0.523	0.508	0.504	0.383	0.488	0.488	0.021
Slovenia (SI)	0.442	0.405	0.505	0.502	0.605	0.463	0.466	0.201	0.454	0.455	0.018
Slovakia (SK)	0.491	0.474	0.544	0.515	0.609	0.473	0.503	0.317	0.494	0.494	0.021
Finland (FI)	0.449	0.395	0.567	0.508	0.687	0.487	0.484	0.133	0.476	0.476	0.019
Sweden (SE)	0.584	0.484	0.541	0.522	0.684	0.321	0.427	0.06	0.471	0.471	0.017

Note: Cell colors indicate acceptance levels—pink for low, yellow for medium, and green for high.

**Table 3:** Ranking of BTQ1–BTQ8 and BT.

Country	Rank BTQ1	Rank BTQ2	Rank BTQ3	Rank BTQ4	Rank BTQ5	Rank BTQ6	Rank BTQ7	Rank BTQ8	Rank BT	RankAv BT ($R_{i}$)
Belgium (BE)	6	7	12	11	19	16	15	7	10	10
Bulgaria (BG)	3	2	5	2	7	4	6	9	2	2
Czechia (CZ)	19	19	24	19	18	17	18	14	19	19
Denmark (DK)	7	11	3	5	2	24	23	26	17	17
Germany (DE)	21	18	14	23	12	25	26	16	25	25
Estonia (EE)	13	14	4	20	13	20	5	23	14	14
Ireland (IE)	12	12	11	7	8	12	9	17	9	9
Greece (EL)	20	15	20	26	16	23	17	19	26	26
Spain (ES)	15	17	22	14	5	8	11	11	11	11
France (FR)	27	27	27	27	22	22	24	21	27	27
Croatia (HR)	18	20	25	21	26	18	19	5	20	20
Italy (IT)	11	5	16	6	21	7	13	3	7	7
Republic of Cyprus (CY)	4	3	13	12	14	10	3	8	5	5
Latvia (LV)	8	6	7	9	11	2	2	18	4	4
Lithuania (LT)	24	24	18	16	23	11	10	15	16	16
Luxembourg (LU)	23	23	23	25	10	19	20	20	23	23
Hungary (HU)	9	13	17	16	25	15	12	4	12	12
Malta (MT)	1	1	1	1	4	1	1	12	1	1
Netherlands (NL)	16	16	6	3	6	21	25	24	18	18
Austria (AT)	22	22	8	24	9	27	27	13	24	24
Poland (PL)	5	9	15	4	24	3	4	1	3	3
Portugal (PT)	17	21	26	22	20	14	21	10	22	22
Romania (RO)	10	10	19	18	27	5	7	2	8	8
Slovenia (SI)	26	25	21	15	17	13	16	22	21	21
Slovakia (SK)	14	8	9	10	15	9	8	6	6	6
Finland (FI)	25	26	2	13	1	6	14	25	13	13
Sweden (SE)	2	4	10	8	3	26	22	27	15	15

**Table 4:** Pearson and Spearman correlation between the B-TOPSIS and the Total positive responses measure for Q1–Q8 questions.

Question	Q1	Q2	Q3	Q4	Q5	Q6	Q7	Q8
Pearson coefficient (B-TOPSIS, Total positive responses)	0.964	0.938	0.943	0.967	0.908	0.972	0.952	0.921
Spearman coefficient (B-TOPSIS, Total positive responses)	0.939	0.927	0.905	0.952	0.854	0.964	0.944	0.944

Legend: Color gradient indicates correlation intensity, from red (lowest) to green (highest).

**Table 5:** Comparison of Total positive responses and B-TOPSIS measure.

Criterion	Total Positive Responses Measure	B-TOPSIS Measure
Response information	Uses only selected positive categories (“very positively” and “somewhat positively”), excluding negative and non-response categories, which leads to partial use of available information.	Incorporates the full response distribution, including all ordinal categories as well as non-responses, ensuring that no information from the survey structure is discarded.
Handling of missing or non-responses	Non-responses are excluded from the calculation, which may distort comparisons when response rates differ across units.	Non-responses are integrated into the belief framework and redistributed in a structured way, preserving comparability across units.
Analytical form and complexity	Relies on simple aggregation, making it easy to compute and interpret, but is limited in capturing distributional features.	Requires belief-structure modelling and distance-based evaluation, increasing methodological complexity but improving informational richness.
Interpretation and use cases	Suitable for quick descriptive benchmarking and reporting where simplicity is prioritised over depth	Suitable for detailed analysis, cross-unit comparison, and uncertainty-aware evaluation where richer information is required.

**Table 6:** Comparative analysis of country rankings for BTQ1–BTQ8 and BT.

BTQ	Description	Top Countries	Bottom Countries	Outliers/Comments	Dominant Acceptance Class
BTQ1	Gathering applicant information	Malta (0.587), Bulgaria (0.540), Cyprus (0.526), Poland (0.525), Latvia (0.502),	France (0.394), Lithuania (0.450), Luxembourg (0.460), Germany (0.465), Greece (0.466)	Malta high outlier, France low outlier	Dominant Medium: 24, Low: 3
BTQ2	Selecting applicants	Malta (0.513), Bulgaria (0.506), Cyprus (0.484), Italy (0.480), Latvia (0.476)	France (0.382), Finland (0.395), Lithuania (0.413), Luxembourg (0.418), Greece (0.444)	Highly correlated with BTQ1	Low: 15, Medium: 12
BTQ3	Allocating tasks	Malta (0.631), Denmark (0.558), Estonia (0.558), Finland (0.567), Latvia (0.547)	France (0.444), Portugal (0.473), Croatia (0.481), Czechia (0.492), Luxembourg (0.493)	Moderate divergence, Malta high outlier	Dominant Medium: 25, Low: 1, High: 1
BTQ4	Processing employee data	Malta (0.612), Denmark (0.536), Italy (0.527), Latvia (0.520), Cyprus (0.511)	France (0.376), Greece (0.381), Germany (0.464), Luxembourg (0.448), Portugal (0.472)	France, Greece low outliers; Malta high outlier	Dominant Medium: 23, Low: 3, High: 1
BTQ5	Improving workers’ safety	Finland (0.687), Denmark (0.685), Sweden (0.684), Malta (0.667), Netherlands (0.641)	Romania (0.523), Hungary (0.568), Croatia (0.566), Poland (0.569), Italy (0.583)	Low dispersion, broad consensus	Dominant High: 17, Medium: 10
BTQ6	Monitoring employees	Malta (0.580), Latvia (0.526), Finland (0.487), Italy (0.481), Hungary (0.449)	Austria (0.319), Sweden (0.321), Germany (0.327), Denmark (0.332), Netherlands (0.364)	High divergence; monitoring-performance cluster	Medium: 14, Low: 13
BTQ7	Performance assessment	Malta (0.592), Latvia (0.519), Cyprus (0.518), Poland (0.512), Estonia (0.510),	Austria (0.384), Germany (0.387), Netherlands (0.399), France (0.403), Denmark (0.414)	Moderate divergence	Dominant Low: 27
BTQ8	Automatic dismissal	Romania (0.383), Poland (0.385), Italy (0.337), Belgium (0.315), Cyprus (0.313)	Sweden (0.060), Denmark (0.090), Finland (0.133), Netherlands (0.139), Austria (0.258)	Highly sensitive; ethical concern dominates	Dominant Low: 21, Medium: 5, High: 1
BT	Overall multidimensional acceptance	Malta (0.556), Bulgaria (0.518), Poland (0.507), Latvia (0.499), Cyprus (0.498),	France (0.401), Greece (0.435), Germany (0.441), Austria (0.443), Luxembourg (0.448)	Aggregation smooths extremes but preserves ranking	Dominant Medium: 22, Low: 5

**Table 7:** Pearson correlation coefficients for BTQ1–BTQ8 and total BT.

	BTQ1	BTQ2	BTQ3	BTQ4	BTQ5	BTQ6	BTQ7	BTQ8	BT
BTQ1	1.000								
BTQ2	0.902	1.000							
BTQ3	0.652	0.575	1.000						
BTQ4	0.735	0.659	0.713	1.000					
BTQ5	0.333	0.121	0.623	0.341	1.000				
BTQ6	0.315	0.406	0.263	0.575	−0.194	1.000			
BTQ7	0.498	0.560	0.424	0.539	−0.089	0.885	1.000		
BTQ8	0.072	0.312	−0.217	0.078	−0.734	0.554	0.445	1.000	
BT	0.794	0.818	0.690	0.855	0.166	0.783	0.834	0.371	1.000

Legend: Color gradient indicates correlation intensity, from red (lowest) to green (highest).

## Data Availability

The data presented in this study are available in Special Eurobarometer SP554: Artificial Intelligence and the Future of Work. Available online: https://data.europa.eu/data/datasets/s3222_101_4_sp554_eng?locale=en (accessed on 1 February 2026), reference numbers [12,37].

## Appendix A

QB8. In general, how positively or negatively do you perceive the use of digital technologies, including Artificial Intelligence, in the workplace for each of the following activities.

**Table A1:** QB8.1. Gathering additional information on applicants for a job.

EU27/Country	Very Positively	Somewhat Positively	Somewhat Negatively	Very Negatively	Don’t Know	Total ‘Positively’	Total ‘Negatively’
EU27	7%	36%	28%	22%	7%	43%	50%
BE	6%	44%	32%	15%	3%	50%	47%
BG	10%	40%	20%	13%	17%	50%	33%
CZ	6%	32%	35%	20%	7%	38%	55%
DK	11%	38%	26%	20%	5%	49%	46%
DE	6%	32%	31%	21%	10%	38%	52%
EE	7%	37%	23%	19%	14%	44%	42%
IE	10%	34%	26%	20%	10%	44%	46%
EL	6%	37%	24%	24%	9%	43%	48%
ES	12%	33%	25%	23%	7%	45%	48%
FR	3%	24%	33%	31%	9%	27%	64%
HR	8%	32%	29%	22%	9%	40%	51%
IT	7%	42%	28%	19%	4%	49%	47%
CY	13%	41%	17%	20%	9%	54%	37%
LV	10%	35%	23%	19%	13%	45%	42%
LT	5%	32%	28%	24%	11%	37%	52%
LU	10%	32%	28%	27%	3%	42%	55%
HU	10%	40%	24%	21%	5%	50%	45%
MT	22%	43%	15%	15%	5%	65%	30%
NL	5%	42%	32%	19%	2%	47%	51%
AT	11%	33%	21%	28%	7%	44%	49%
PL	7%	47%	26%	14%	6%	54%	40%
PT	6%	34%	23%	21%	16%	40%	44%
RO	8%	38%	26%	18%	10%	46%	44%
SI	7%	30%	32%	27%	4%	37%	59%
SK	8%	37%	30%	19%	6%	45%	49%
FI	4%	34%	32%	24%	6%	38%	56%
SE	16%	52%	17%	11%	4%	68%	28%

Source: [12,37].

**Table A2:** QB8.2. Selecting applicants for a job.

EU27/Country	Very Positively	Somewhat Positively	Somewhat Negatively	Very Negatively	Don’t Know	Total ‘Positively’	Total ‘Negatively’
EU27	5%	31%	32%	25%	7%	36%	57%
BE	4%	38%	36%	19%	3%	42%	55%
BG	9%	35%	23%	17%	16%	44%	40%
CZ	4%	28%	37%	26%	5%	32%	63%
DK	6%	32%	32%	24%	6%	38%	56%
DE	4%	29%	32%	25%	10%	33%	57%
EE	4%	30%	29%	23%	14%	34%	52%
IE	8%	28%	30%	25%	9%	36%	55%
EL	6%	34%	24%	28%	8%	40%	52%
ES	8%	28%	29%	29%	6%	36%	58%
FR	3%	22%	34%	33%	8%	25%	67%
HR	5%	28%	31%	27%	9%	33%	58%
IT	7%	36%	33%	20%	4%	43%	53%
CY	13%	32%	22%	25%	8%	45%	47%
LV	9%	30%	27%	21%	13%	39%	48%
LT	4%	27%	30%	29%	10%	31%	59%
LU	5%	28%	34%	30%	3%	33%	64%
HU	8%	33%	25%	29%	5%	41%	54%
MT	13%	39%	22%	21%	5%	52%	43%
NL	4%	32%	37%	25%	2%	36%	62%
AT	10%	28%	24%	34%	4%	38%	58%
PL	5%	36%	33%	20%	6%	41%	53%
PT	4%	29%	25%	27%	15%	33%	52%
RO	6%	34%	30%	21%	9%	40%	51%
SI	7%	24%	32%	33%	4%	31%	65%
SK	6%	36%	30%	21%	7%	42%	51%
FI	2%	27%	34%	32%	5%	29%	66%
SE	7%	38%	32%	20%	3%	45%	52%

Source: [12,37].

**Table A3:** QB8.3. Allocating tasks to workers or managing their working schedules and shifts.

EU27/Country	Very Positively	Somewhat Positively	Somewhat Negatively	Very Negatively	Don’t Know	Total ‘Positively’	Total ‘Negatively’
EU27	8%	41%	27%	17%	7%	49%	44%
BE	8%	47%	30%	13%	2%	55%	43%
BG	12%	42%	19%	12%	15%	54%	31%
CZ	6%	40%	31%	18%	5%	46%	49%
DK	14%	46%	22%	13%	5%	60%	35%
DE	6%	44%	26%	13%	11%	50%	39%
EE	12%	42%	18%	12%	16%	54%	30%
IE	12%	42%	21%	15%	10%	54%	36%
EL	8%	42%	21%	19%	10%	50%	40%
ES	13%	36%	23%	22%	6%	49%	45%
FR	3%	32%	34%	23%	8%	35%	57%
HR	7%	34%	31%	19%	9%	41%	50%
IT	8%	45%	27%	16%	4%	53%	43%
CY	14%	40%	17%	21%	8%	54%	38%
LV	12%	39%	23%	12%	14%	51%	35%
LT	8%	42%	23%	17%	10%	50%	40%
LU	6%	44%	27%	20%	3%	50%	47%
HU	11%	41%	23%	19%	6%	52%	42%
MT	27%	46%	11%	11%	5%	73%	22%
NL	8%	52%	28%	9%	3%	60%	37%
AT	18%	38%	17%	19%	8%	56%	36%
PL	7%	45%	28%	14%	6%	52%	42%
PT	5%	36%	23%	21%	15%	41%	44%
RO	8%	39%	28%	16%	9%	47%	44%
SI	11%	40%	24%	21%	4%	51%	45%
SK	10%	46%	25%	12%	7%	56%	37%
FI	10%	54%	21%	9%	6%	64%	30%
SE	12%	43%	27%	13%	5%	55%	40%

Source: [12,37].

**Table A4:** QB8.4. Collecting, processing, and storing workers’ personal data.

EU27/Country	Very Positively	Somewhat Positively	Somewhat Negatively	Very Negatively	Don’t Know	Total ‘Positively’	Total ‘Negatively’
EU27	8%	36%	28%	21%	7%	44%	49%
BE	9%	42%	31%	16%	2%	51%	47%
BG	12%	43%	18%	12%	15%	55%	30%
CZ	9%	37%	29%	20%	5%	46%	49%
DK	15%	38%	26%	16%	5%	53%	42%
DE	5%	32%	33%	20%	10%	37%	53%
EE	8%	34%	25%	19%	14%	42%	44%
IE	13%	36%	23%	17%	11%	49%	40%
EL	2%	26%	28%	34%	10%	28%	62%
ES	14%	35%	23%	22%	6%	49%	45%
FR	3%	21%	35%	34%	7%	24%	69%
HR	8%	37%	28%	20%	7%	45%	48%
IT	10%	45%	25%	16%	4%	55%	41%
CY	13%	40%	17%	23%	7%	53%	40%
LV	13%	36%	20%	19%	12%	49%	39%
LT	7%	40%	25%	18%	10%	47%	43%
LU	6%	33%	30%	26%	5%	39%	56%
HU	11%	39%	23%	22%	5%	50%	45%
MT	26%	41%	14%	13%	6%	67%	27%
NL	11%	47%	27%	12%	3%	58%	39%
AT	11%	32%	21%	31%	5%	43%	52%
PL	8%	49%	23%	13%	7%	57%	36%
PT	7%	32%	25%	21%	15%	39%	46%
RO	8%	38%	27%	19%	8%	46%	46%
SI	13%	35%	27%	21%	4%	48%	48%
SK	9%	42%	25%	17%	7%	51%	42%
FI	7%	44%	27%	17%	5%	51%	44%
SE	12%	40%	27%	17%	4%	52%	44%

Source: [12,37].

**Table A5:** QB8.5. Improving workers’ safety and security.

EU27/Country	Very Positively	Somewhat Positively	Somewhat Negatively	Very Negatively	Don’t Know	Total ‘Positively’	Total ‘Negatively’
EU27	20%	47%	16%	9%	8%	67%	25%
BE	18%	52%	19%	9%	2%	70%	28%
BG	25%	39%	12%	8%	16%	64%	20%
CZ	19%	47%	17%	10%	7%	66%	27%
DK	35%	43%	11%	5%	6%	78%	16%
DE	20%	50%	14%	7%	9%	70%	21%
EE	18%	49%	9%	9%	15%	67%	18%
IE	25%	43%	14%	8%	10%	68%	22%
EL	21%	45%	14%	11%	9%	66%	25%
ES	31%	41%	14%	8%	6%	72%	22%
FR	15%	47%	17%	11%	10%	62%	28%
HR	16%	42%	22%	12%	8%	58%	34%
IT	17%	49%	19%	11%	4%	66%	30%
CY	25%	44%	10%	14%	7%	69%	24%
LV	20%	44%	14%	7%	15%	64%	21%
LT	14%	48%	15%	12%	11%	62%	27%
LU	22%	50%	16%	7%	5%	72%	23%
HU	16%	47%	18%	14%	5%	63%	32%
MT	34%	42%	8%	10%	6%	76%	18%
NL	25%	51%	15%	5%	4%	76%	20%
AT	27%	43%	11%	11%	8%	70%	22%
PL	11%	52%	21%	9%	7%	63%	30%
PT	19%	40%	13%	13%	15%	59%	26%
RO	11%	39%	25%	16%	9%	50%	41%
SI	25%	42%	16%	13%	4%	67%	29%
SK	20%	48%	17%	8%	7%	68%	25%
FI	33%	52%	8%	3%	4%	85%	11%
SE	32%	50%	9%	3%	6%	82%	12%

Source: [12,37].

**Table A6:** QB8.6. Monitoring workers.

EU27/Country	Very Positively	Somewhat Positively	Somewhat Negatively	Very Negatively	Don’t Know	Total ‘Positively’	Total ‘Negatively’
EU27	5%	26%	35%	28%	6%	31%	63%
BE	4%	30%	41%	23%	2%	34%	64%
BG	8%	36%	25%	15%	16%	44%	40%
CZ	4%	27%	41%	24%	4%	31%	65%
DK	3%	16%	34%	42%	5%	19%	76%
DE	2%	12%	39%	40%	7%	14%	79%
EE	4%	23%	32%	28%	13%	27%	60%
IE	8%	32%	28%	23%	9%	40%	51%
EL	3%	17%	34%	40%	6%	20%	74%
ES	11%	30%	30%	22%	7%	41%	52%
FR	2%	17%	39%	35%	7%	19%	74%
HR	4%	28%	33%	27%	8%	32%	60%
IT	6%	37%	34%	19%	4%	43%	53%
CY	11%	33%	23%	26%	7%	44%	49%
LV	11%	36%	26%	14%	13%	47%	40%
LT	5%	33%	33%	19%	10%	38%	52%
LU	6%	26%	34%	31%	3%	32%	65%
HU	5%	35%	29%	26%	5%	40%	55%
MT	20%	43%	20%	13%	4%	63%	33%
NL	3%	17%	42%	36%	2%	20%	78%
AT	7%	17%	23%	49%	4%	24%	72%
PL	6%	44%	29%	14%	7%	50%	43%
PT	5%	31%	26%	23%	15%	36%	49%
RO	8%	39%	28%	16%	9%	47%	44%
SI	8%	31%	34%	23%	4%	39%	57%
SK	7%	34%	32%	21%	6%	41%	53%
FI	5%	37%	37%	16%	5%	42%	53%
SE	2%	15%	36%	44%	3%	17%	80%

Source: [12,37].

**Table A7:** QB8.7. Assessing workers’ performance.

EU27/Country	Very Positively	Somewhat Positively	Somewhat Negatively	Very Negatively	Don’t Know	Total ‘Positively’	Total ‘Negatively’
EU27	6%	30%	34%	23%	7%	36%	57%
BE	5%	36%	37%	19%	3%	41%	56%
BG	8%	35%	25%	15%	17%	43%	40%
CZ	4%	34%	36%	21%	5%	38%	57%
DK	4%	28%	34%	30%	4%	32%	64%
DE	2%	21%	38%	30%	9%	23%	68%
EE	7%	37%	23%	15%	18%	44%	38%
IE	10%	33%	28%	19%	10%	43%	47%
EL	6%	36%	26%	24%	8%	42%	50%
ES	12%	31%	30%	21%	6%	43%	51%
FR	2%	24%	38%	28%	8%	26%	66%
HR	5%	32%	32%	22%	9%	37%	54%
IT	6%	38%	34%	18%	4%	44%	52%
CY	12%	41%	20%	20%	7%	53%	40%
LV	10%	36%	25%	15%	14%	46%	40%
LT	6%	36%	30%	17%	11%	42%	47%
LU	6%	33%	34%	24%	3%	39%	58%
HU	8%	40%	24%	22%	6%	48%	46%
MT	22%	42%	17%	13%	6%	64%	30%
NL	4%	21%	42%	30%	3%	25%	72%
AT	8%	25%	24%	40%	3%	33%	64%
PL	7%	41%	31%	14%	7%	48%	45%
PT	5%	31%	27%	24%	13%	36%	51%
RO	7%	39%	28%	16%	10%	46%	44%
SI	9%	32%	31%	24%	4%	41%	55%
SK	8%	39%	29%	17%	7%	47%	46%
FI	5%	37%	35%	17%	6%	42%	52%
SE	4%	29%	36%	27%	4%	33%	63%

Source: [12,37].

**Table A8:** QB8.8. Automatically firing workers.

EU27/Country	Very Positively	Somewhat Positively	Somewhat Negatively	Very Negatively	Don’t Know	Total ‘Positively’	Total ‘Negatively’
EU27	3%	13%	24%	54%	6%	16%	78%
BE	3%	16%	32%	47%	2%	19%	79%
BG	4%	14%	23%	45%	14%	18%	68%
CZ	2%	10%	29%	55%	4%	12%	84%
DK	-	2%	12%	82%	4%	2%	94%
DE	3%	11%	22%	57%	7%	14%	79%
EE	2%	5%	21%	61%	11%	7%	82%
IE	4%	11%	18%	59%	8%	15%	77%
EL	1%	7%	26%	61%	5%	8%	87%
ES	4%	12%	24%	54%	6%	16%	78%
FR	1%	6%	25%	62%	6%	7%	87%
HR	3%	15%	31%	43%	8%	18%	74%
IT	3%	19%	32%	43%	3%	22%	75%
CY	6%	19%	18%	49%	8%	25%	67%
LV	3%	10%	19%	56%	12%	13%	75%
LT	1%	10%	25%	52%	12%	11%	77%
LU	2%	9%	21%	64%	4%	11%	85%
HU	5%	19%	25%	47%	4%	24%	72%
MT	3%	10%	29%	54%	4%	13%	83%
NL	1%	4%	17%	76%	2%	5%	93%
AT	5%	18%	13%	61%	3%	23%	74%
PL	5%	28%	24%	38%	5%	33%	62%
PT	2%	14%	21%	49%	14%	16%	70%
RO	7%	21%	28%	36%	8%	28%	64%
SI	1%	6%	25%	64%	4%	7%	89%
SK	3%	16%	30%	45%	6%	19%	75%
FI	-	4%	17%	76%	3%	4%	93%
SE	1%	2%	6%	89%	2%	3%	95%

Source: [12,37].

## Appendix B

**Table A9:** QB8. In general, how positively or negatively do you perceive the use of digital technologies, including Artificial Intelligence, in the workplace for each of the following activities? (% Total Positively).

	QB8.1	QB8.2	QB8.3	QB8.4	QB8.5	QB8.6	QB8.7	QB8.8
EU27	43	36	49	44	67	31	36	16
Gender								
Man	46	38	51	45	70	32	39	16
Women	40	34	47	43	65	30	34	16
Age								
15–24	53	46	62	57	77	44	49	23
25–39	47	40	55	40	69	35	41	18
40–54	45	37	52	45	69	32	39	17
55+	35	30	40	37	62	24	28	12
Socio-professional category								
Self-employed	47	42	54	49	70	36	41	17
Managers	51	41	60	51	76	32	40	15
Other white collars	51	43	54	53	73	38	43	19
Manual workers	41	34	47	41	64	32	36	19
Home persons	36	29	44	40	53	28	35	16
Unemployment	37	27	44	35	66	28	29	12
Subjective urbanization								
Rural village	39	31	42	39	62	28	33	15
Small/middle size town	43	36	51	45	68	32	36	16
Large town	46	40	53	49	71	33	39	17
Sector of employment								
Agriculture forestry and fishing	38	28	40	38	63	33	32	16
Manufacturing	46	41	54	46	69	36	40	20
Logistic	52	43	59	52	69	42	46	23
Service	47	39	53	47	70	33	40	17
Public sector	47	38	54	47	71	31	36	16
Awareness of the use that your employer makes of digital technologies, including AI								
Aware	56	46	63	56	78	41	48	23
Unaware	31	27	36	32	56	22	25	11

Source: [12,37].
